# Effects of infant age and sex, and maternal parity on the interaction of lactation with infant feeding development in chimpanzees

**DOI:** 10.1371/journal.pone.0272139

**Published:** 2022-08-04

**Authors:** Iulia Bădescu, David P. Watts, Cassandra Curteanu, Kelly J. Desruelle, Daniel W. Sellen

**Affiliations:** 1 Département d’anthropologie, Université de Montréal, Montréal, QC, Canada; 2 Department of Anthropology, Yale University, New Haven, CT, United States of America; 3 Department of Anthropology, University of Toronto, Toronto, ON, Canada; Sichuan University, CHINA

## Abstract

The interaction between infant feeding and maternal lactational physiology influences female inter-birth intervals and mediates maternal reproductive trade-offs. We investigated variation in feeding development in 72 immature wild chimpanzees (*Pan troglodytes schweinfurthii*) at Ngogo, Kibale National Park, Uganda, and made inferences about maternal lactation over the course of infancy. We compared the percentage (%) of time that mothers nursed infants as a function of infant age and assessed how hourly rates and bout durations of nursing and foraging varied in association with differences in offspring age, sex, and maternal parity. Nursing % times, rates and durations were highest for infants ≤ 6 months old but did not change significantly from 6 months to 5 years old. Nursing continued at a decreasing rate for some 5- to 7-year-olds. Infants ≤ 6 months old foraged little. Foraging rates did not change after 1 year old, but foraging durations and the % time devoted to foraging increased with age. Independent foraging probably became a dietary requirement for infants at 1 year old, when their energy needs may have surpassed the available milk energy. Infants spent as much time foraging by the time they were 4 to 5 years old as adults did. No sex effect on infant nursing or foraging was apparent, but infants of primiparous females had higher foraging rates and spent more time foraging than the infants of multiparous females did. Although no data on milk composition were collected, these findings are consistent with a working hypothesis that like other hominoids, chimpanzee mothers maintained a fixed level of lactation effort over several years as infants increasingly supplemented their growing energy, micronutrient and hydration needs via independent foraging. Plateauing lactation may be a more widespread adaptation that allows hominoid infants time to attain the physiology and skills necessary for independent feeding, while also providing them with a steady dietary base on which they could rely consistently through infancy, and enabling mothers to maintain a fixed, predictable level of lactation effort.

## Introduction

Infant feeding development (or infant nutritional development) in mammals is the transition from complete dependence on maternal milk to nutritional independence [1–3]. Stages of feeding development include exclusive suckling; transitional feeding, which starts when infants first consume non-milk food; and weaning, which ends after the last nursing bout with milk transfer [1,2]. Comfort nursing (without milk transfer), in which immature individuals continue to make non-nutritive nipple contacts after the completion of lactation [4,5], may also occur. When it does, it leads to different physiological and behavioral weaning ages unless nutritional and behavioral weaning occur at the same time, as is often the case [4,6]. A physiologically weaned individual has stopped ingesting maternal milk because milk transfer has ended but is not yet behaviorally weaned if they still make nipple contacts. A behaviorally weaned individual has stopped making nipple contacts but might already have been physiologically weaned.

Changes in suckling and foraging by infants as they age influence the scheduling and duration of feeding development stages and affect early-life rates of growth and survival [3,7]. Furthermore, since nursing and foraging behaviors reflect the distribution of infants’ dietary needs for milk and non-milk foods, changes in infant feeding behavior illuminate the interaction between infant nutritional requirements over the course of development and maternal lactational physiology. This is because milk synthesis is largely a maternal physiological response to mechanical nipple stimulation by the suckling infant [8–13]. The interaction between infant feeding behavior and maternal lactational physiology can influence inter-birth intervals and mediate important maternal trade-offs between current and future reproduction and number versus quality of offspring [3,14–17].

In primates, the time spent independently foraging by immature individuals generally increases with increasing age [18–25], which is a proxy for size, development of ecological competency, and total metabolic needs. Cross-species data show that, counter-intuitively, the length of time spent nursing can either decrease, increase, or remain stable through the majority of infancy until it finally decreases and stops at behavioral weaning. When nursing gradually decreases with increasing infant age (e.g. olive baboon, *Papio anubis*: [26]; rhesus macaque, *Macaca mulatta*: [9,10]; blue monkey, *Cercopithecus mitis stuhlmanni*: [24]; vervet monkey, *Chlorocebus pygerythrus*: [27]; mountain gorilla, *Gorilla beringei*: [28]; chimpanzee, *Pan troglodytes schweinfurthii* at Gombe: [29], and Mahale: [23]), maternal lactation effort should decline over time because females reduce lactational investment as their offspring grow and develop foraging competence [5,10,30,31].

Alternatively, when infants nurse progressively more frequently and/or for longer per day as they age (e.g. chimpanzee, *Pan troglodytes schweinfurthii* at Kanyawara: [22]), maternal lactation effort may increase with infant age to support the increasing energetic needs of larger, developing infants [18,32]. Increasing nursing behavior may thus reflect increased maternal milk synthesis over time, because while infants spend more time foraging as they gradually attain independent feeding abilities, they also continue to obtain much of the energy they need for growth and maintenance from milk.

Finally, nursing behaviors may remain relatively constant through most of infancy (e.g. Japanese macaque, *Macaca fuscata*: [33]; rhesus macaque, *Macaca mulatta*: [11]; yellow baboon, *Papio cynocephalus*: [18]; gelada, *Theropithecus gelada*: [34]; chimpanzee, *Pan troglodytes schweinfurthii* at Gombe: [21] and Ngogo: this study; orangutan, *Pongo pygmaeus wurmbii*: [35]), which may reflect a regular rate of maternal milk synthesis. Milk transfer rates in such cases presumably remain constant and females maintain steady lactational investment while offspring attain independent feeding abilities, with infants meeting their additional growth-, developmental-, and size-related energy and nutrient needs through increased foraging during the transition to nutritional independence [1,3,32,35–38]. Offspring could thus rely on a stable and predictable milk supply through most of infancy. Anticipating the milk supply would allow infants to compensate for gaps between their energy needs and the energy available from milk by foraging independently.

The suckling efficiency of infants, however, can also increase with age as infants develop the ability to coordinate suckling rate with swallowing and respiration. Consequently, while time spent nursing can change or remain constant as infants age, the amount of milk transfer can increase or remain the same [39]. Similarly, the composition and nutrient density of milk secreted may change with infant age and development and is rarely investigated below gross nutrient composition level [40].

The age at which infants first ingest non-milk foods, and thus the age at which transitional feeding begins, does not necessarily overlap with the age at which infants must regularly supplement their diet with non-milk foods because they have physiologically outgrown the energy available to them from maternal milk [3,38]. For instance, non-human ape infants may not physiologically need to feed on non-milk foods until around 1 year old, even though they often sample non-milk foods several months before this age [3,4,38]. This point in feeding development is also different from the age at which immature primates are developmentally ready to rely more heavily on adult foods, which could help predict when survival is possible–though potentially still unlikely–without the continuing nutritional support of maternal milk [23,36,41,42]. Infant chimpanzees, for example, may start to rely more heavily on solid foods at 3 years old [23,41], and they spend as much time as adults foraging independently by 4 to 5 years old, which is also the age range within which behavioral and physiological weaning usually occurs [4,21,22]. Indeed, orphan chimpanzees in the wild can only survive without nursing after 3 years old, and their survival chances decline if their mother dies before they reach age 5 [43,44]. Thus, while the point at which primate infants quantitatively increase their nutritional dependence on solid food and reduce their dependence on maternal milk may happen sometime before physiological weaning, continued nursing and maternal milk transfer to infants after this point is adaptive for females and their offspring [1,3,36,42].

Considerable intra-specific variation overlies species-typical patterns in infant feeding behavior. Variation in infant feeding patterns can lead to differential rates of growth and development and to variation in the length of infant dependency on mothers [3,16,36]. Several factors are associated with variation in nursing and milk intake rates [40], including maternal health condition [45–49], parity and reproductive experience [13,49–51], quantity and quality of milk produced [9,51–54]; maternal activities outside of infant care [18,34]; alloparental care [55,56]; infant sex [13,51,57–59], hunger [39,60], age, and nursing efficiency [61]; and variation in food availability [25,62]. Solid food intake rates of immature individuals may vary with age [18–25,63] and sex [24,64]; the extent to which others share food with them [65–67]; and variation in the ease of processing different types of food and temporal variation in food availability [25,62,67].

Data on infant diets from additional sites provide insights into how life history theory explains intraspecific variation in lactational effort and feeding ontogeny and help reveal which associated traits are more plastic or more constrained [1,38,68]. Wild chimpanzee infant feeding behavior has been described at several sites, but detailed descriptions of nursing and the development of independent foraging have not previously been available for wild chimpanzees (*Pan troglodytes schweinfurthii*) at Ngogo, Uganda. Previous fecal stable nitrogen isotope analyses indicated a physiological weaning age at Ngogo of 4 to 4.5 years old [4]. While many infants were also behaviorally weaned by this age, several showed prolonged comfort nursing and were not behaviorally weaned until 5.5 to 6 years old, or even 7 to 8 years old, based on observations of nipple contacts [4,6]. Males were behaviorally, and probably physiologically, weaned later than females [6]. Stable carbon and nitrogen isotope data showed that the proportions of milk to non-milk food in the diets of infants gradually decreased through most of infancy, consistent with the occurrence of a gradual physiological weaning process over several years [4]. However, data on how nursing and independent foraging behaviors are expressed through infancy are needed to understand whether this gradual physiological weaning process results from reduced maternal lactational investment over the course of infancy or from infants relying progressively more on independent foraging while maintaining their reliance on milk [6]. Here we report new results of analyses on feeding development in the Ngogo chimpanzee community using behavioral data on nursing and foraging by 72 immature individuals ranging from 0 to 9 years old. To delineate variation in infant feeding and to infer how maternal lactation changed over the course of infancy, we assessed how infant age, sex and maternal parity affected the percentage (%) of time that infants spent nursing and independently foraging and the rates and durations of nursing and foraging bouts.

## Methods

### Study site and species

The Ngogo study site, in the center of Kibale National Park, Uganda, comprises about 35 km^2^ of mostly old growth forest and also includes colonizing forest, grasslands, and swamp areas of *Acanthus pubescens* and *Cyperus papyrus* [69,70]. Dry seasons are separated by rainy seasons that occur from March to June and September to November [70,71].

The Ngogo chimpanzee community numbered between 199 and 207 individuals, including 54 to 57 adult females, 26 to 33 adult males, 30 to 34 juvenile and adolescent females, 33 to 42 juvenile and adolescent males, and 40 to 53 infants during the study periods. The community was in the process of undergoing a permanent fission by the 2018 study period [72]. Chimpanzees at Ngogo eat primarily fruit, especially a fig species, *Ficus mucuso*, as well as relying heavily on leaves, and to a lesser extent on other plant parts, such as pith and stems [70,73]. They also hunt and eat a variety of mammals, particularly red colobus monkeys (*Procolobus badius*) [74–76]. Females at Ngogo do not form dominance hierarchies [77]. Female conflicts are generally rare, and when they do occur, clear or consistent outcomes, with decided winners and losers, are uncommon [77–79].

### Study subjects

We studied 72 immature chimpanzees aged 0 to 9 years old. None had younger maternal siblings–that is, their mothers did not have new infants during the data collection period. These 72 individuals were offspring of 56 adult females, as 16 adult females contributed two offspring to our dataset. We refer to individuals aged 0 to ≤ 5 years old as “infants” [80]. Data on the first appearance of infants and on the last appearance of their mothers prior to births were used to estimate ages of study subjects. Estimated ages varied mostly from one or several days to within a month. For two individuals, birth date estimates were within several months and were partly based on comparisons of their nutritional, physical and social independence relative to other infants with precise age estimates. Data collectors could individually recognize the chimpanzees. Researchers learned to recognize individual chimpanzees based on distinguishing facial features and color patterns, scars, ear knicks, missing fingers, limbs or eyes, and hair color and distribution on the head or body.

### Data collection

Data were collected on directly observed daytime feeding of immature chimpanzees by IB from January to March 2013 and September 2013 to June 2014, and by IB, CC and KJD from January to April 2018 using focal animal sampling [81]. IB trained CC and KJD in the field to help ensure consistency and reliability in data collection between the three observers. During 1-hour samples, observers continuously recorded the frequency and duration focal subjects spent nursing (i.e., making nipple contact) and foraging (i.e., ingesting non-milk or solid foods). We excluded times when study subjects were on their mothers’ ventrums with their faces not visible. Focal subjects were foraging when they independently looked for, picked, bit, chewed and ingested food that they acquired themselves. We excluded exploratory mouthing or handling of vegetation that did not lead to ingestion. Focal samples were terminated if the subject was out of view for more than 10 minutes. When feeding bouts lasted beyond the end of the 1-hour focal sample, observers tried to record the end time of the bout. Initial selection of focal subjects on a daily basis was either random or aimed at those individuals for whom data were particularly needed. After completing a sample, observers chose another individual from among those present and subsequently aimed to cycle through all individuals present in the same order for the rest of the day. Observers attempted to collect behavioral data that were evenly distributed between mornings (7am-12pm) and afternoons (12pm-5:30pm) to minimize the potential effects of diurnal variation in activities. We obtained a total of 1245.2 focal sampling hours and a mean of 12.4 hours per focal subject (Table 1), not including time that subjects were out of view. Focal follows were done over a mean of 8 ± 4 (SD) different days per study subject by age category.

**Table 1:** Study subjects and focal sampling hours.

Infant age (years)	Number of focal individuals (female, male)	Mean number of focal hours (SD)
0–0.5	14 (9, 4, 1 sex unknown)	12.05 (6.97)
> 0.5–1	10 (6, 4)	13.37 (6.59)
> 1–2	19 (11, 8)	13.14 (5.60)
> 2–3	22 (9, 13)	11.86 (5.52)
> 3–4	17 (9, 8)	11.58 (5.42)
> 4–5	7 (4, 3)	14.38 (5.95)
> 5–6	4 (1, 3)	9.50 (3.19)
> 6–7	4 (2, 2)	16.64 (13.95)
> 7–8	2 (0, 2)	11.88 (6.61)
> 8–9	1 (0, 1)	6.27 (na)
**Total**	**100 (51, 48, 1 sex unknown)**	**12.39 (6.51)**

All aspects of this research were approved by the Uganda Wildlife Authority (UWA), Uganda National Council for Science and Technology (UNCST), Makerere University in Uganda, the University of Toronto’s Office of Research Ethics and Environmental Health and Safety, and Université de Montréal’s Comité de déontologie de l’expérimentation sur les animaux (CDEA). Additional information regarding the ethical, cultural, and scientific considerations specific to inclusivity in global research is included in the Supporting Information (S1 Checklist).

### Data analyses

We separated the behavioral data from birth to year 1 into two 6-month increments (0 to ≤ 6 months old, > 6 months to ≤ 1 year old), as the end of exclusive suckling and beginning of transitional feeding in apes can occur before infants are 1 year old [1,3,4,63]. We pooled behavioral data on older individuals into 1-year increments (> 1 to 2 years old, > 2 to 3 years old, etc.; Table 1). To determine the amount of time that immature chimpanzees spent nursing or foraging, we calculated behavioral % times for each study subject by dividing the total number of hours they spent nursing or foraging by the total number of focal sampling hours at each age category, multiplied by 100. Nursing and foraging behaviors were considered distinct bouts when separated by at least 1 minute [9,27,28]. To obtain hourly behavioral rates, we divided bout frequencies of nursing or foraging by the total focal sampling hours at each age category for each study subject. We used nursing rates because they have been shown to correlate positively with milk synthesis and negatively with the resumption of ovulation [10,27,36]. To determine mean nursing bout durations, we included only complete bouts for which we saw both beginnings and endings. We obtained mean nursing bout durations for each age category by subtracting the onset of nursing from the end time for a given bout, adding all bout durations, and dividing the sum by the number of nursing bouts for each infant. Foraging bout durations varied much more than those of nursing bouts and we had fewer complete foraging bouts (observed from beginning to end), than incomplete bouts (either beginning, end, or both not seen). Complete foraging bouts were skewed towards those with the shortest durations. To determine mean foraging bout durations for each age category, we included both complete and incomplete bouts and subtracted the onset of foraging (or time when observation of foraging started) from the end time (or time when observation ended) for a given bout, adding all bout durations, and dividing by the number of foraging bouts for each infant.

#### Statistical analyses

We conducted Generalized Estimating Equations (GEE) analyses [82–84] to evaluate how the % time spent nursing and foraging and the rate and duration of nursing and foraging bouts varied in relation age category, sex (male or female), and maternal parity (primiparous or multiparous). We conducted a first set of GEEs on all immature chimpanzees, 0 to ≤ 9 years old. Of the 72 individuals, 23 contributed data to multiple age categories. Our total sample size used in the first set of GEEs was thus 100 individuals by age category (Table 1).

As a further evaluation of how feeding varied during infancy, we conducted a second set of GEEs on infants > 6 months to ≤ 5 years old. We excluded subjects of the youngest age category (0 to ≤ 6 months old), because adjustment in lactation, nursing, and infant metabolic physiology shortly after birth [12,85–87] could lead to differences in nursing between the youngest infants and those infants in older age categories. We also excluded subjects from the age categories > 5 years old because most chimpanzees are physiologically and behaviorally weaned at Ngogo by 4.5 years old, and we expected that most nipple contacts after age 5 would be non-nutritive [4]. We included 55 infants aged > 6 months to ≤ 5 years old; 16 of these contributed data to multiple age categories, so the total sample size for the second set of GEEs was 75 infants by age category.

We included infant and mother identities as the grouping structure in the analyses to control for repeated measurements of the same subjects. We ran statistical analyses using SPSS version 27, with alpha set at 0.05, and applied a Bonferroni correction to the resulting p values to account for multiple testing of the same sample.

## Results

### Nursing

**Fig 1:**
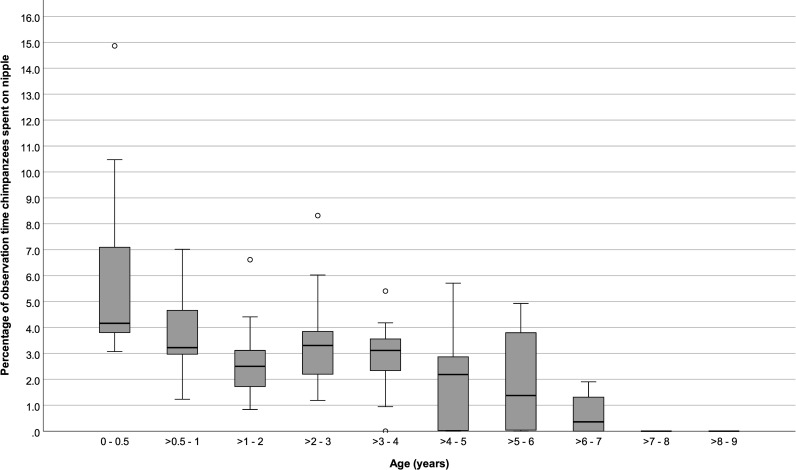
Percentage of focal observation time that immature chimpanzees spent nursing at different ages. Box plots show median values (solid horizontal lines), 25^th^ and 75^th^ percentile values (box boundaries), highest and lowest values (whiskers) and outliers (circles).

**Fig 2:**
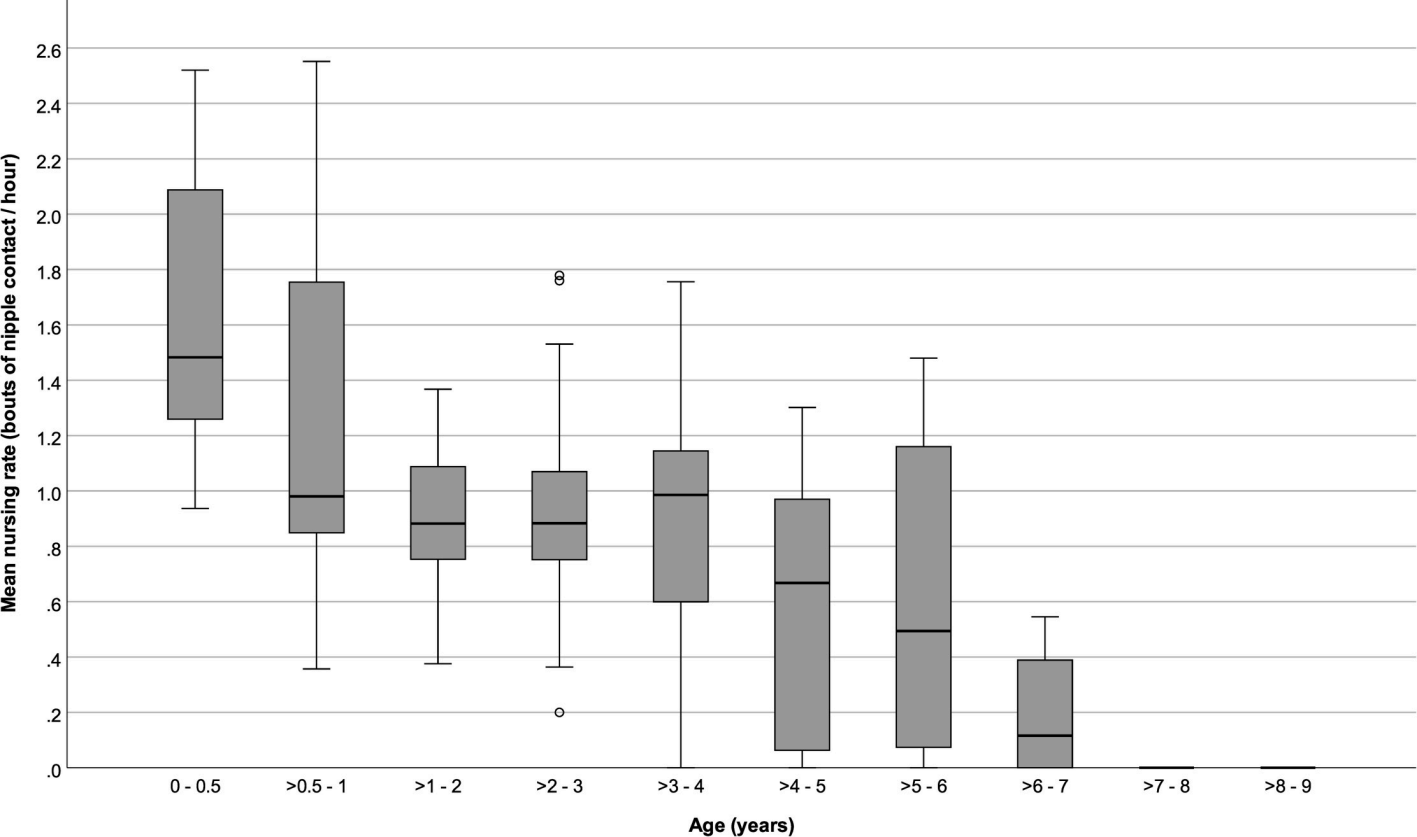
Average nursing rates of immature chimpanzees at different ages. Box plots show median values (solid horizontal lines), 25^th^ and 75^th^ percentile values (box boundaries), highest and lowest values (whiskers) and outliers (circles).

**Fig 3:**
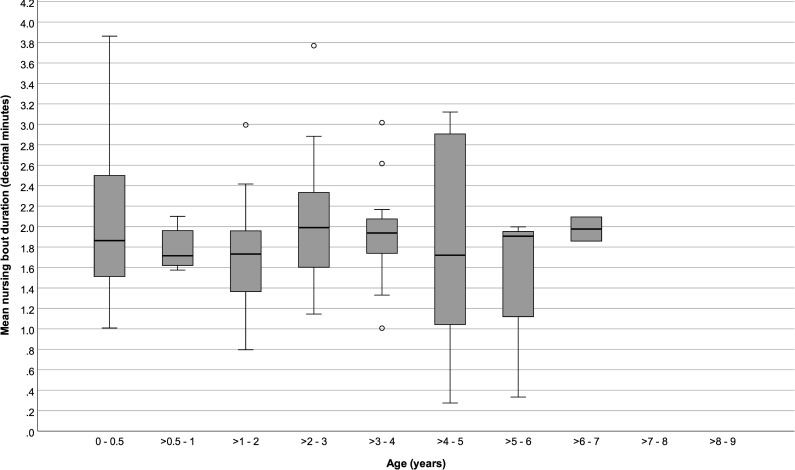
Average nursing bout durations of immature chimpanzees at different ages. Box plots show median values (solid horizontal lines), 25^th^ and 75^th^ percentile values (box boundaries), highest and lowest values (whiskers) and outliers (circles).

Infants ≤ 6 months old spent 5.85 (SD: ± 3.4) % of their time suckling, and nursed on average for 1.63 (± 0.51) bouts per hour and for 2.03 (SD: ± 0.73) minutes per nursing bout. After > 6 months and until ≤ 4 years old, mean time spent nursing and mean nursing bout rates stayed around 3 (± 0.51) % and 1.00 (± 0.44) bout per hour, and nursing durations remained close to 2.00 (± 0.50) minutes per bout. Nursing started to decrease after 4 years old, although it sometimes continued until offspring were 7 years old (Figs 1 to 3).

Percent time spent nursing and nursing hourly rates were higher for younger infants and decreased with age (GEE: P < 0.001 and < 0.001, respectively; Table 2), but average nursing bout durations did not vary with age (GEE: *P* = 0.84). Repeating the analyses only among infants > 6 months to ≤ 5 years old showed no significant changes in nursing % times, rates, or durations with age (GEE: *P* = 0.64, *P* = 0.25, P = 0.95, respectively; Table 3). Neither the sex of the infants nor the parity of their mothers predicted variation in nursing parameters (Tables 2 and 3).

**Table 2:** Generalized Estimating Equations (GEEs) for correlations between feeding parameters of chimpanzees 0 to 9 years old and infant age, sex, and maternal parity.

Infant feeding parameter	Infant or Maternal Characteristic	β	SE	95% Wald confidence interval		Hypothesis Test	
				Lower	Upper	Wald χ2 (df = 1)	*P*
Percent time spent nursing	Infant age	-0.54	0.11	-0.75	-0.33	25.03	**0.000**
	Infant sex	0.02	0.36	-0.69	0.73	0.004	1.00
	Maternal parity	-0.70	0.44	-1.56	0.16	2.56	0.22
Nursing hourly rate	Infant age	-0.15	0.02	-0.20	-0.11	53.01	**0.000**
	Infant sex	0.01	0.08	-0.15	0.18	0.03	1.00
	Maternal parity	-0.09	0.09	-0.27	0.08	1.06	0.61
Nursing bout duration	Infant age	-0.001	0.001	-0.002	0.001	0.66	0.84
	Infant sex	0.000	0.002	-0.005	0.004	0.04	1.00
	Maternal parity	-0.004	0.002	-0.008	0.001	2.29	0.26
Percent time spent foraging	Infant age	9.30	1.41	6.54	12.06	43.63	**0.000**
	Infant sex	4.31	3.66	-2.86	11.48	1.39	0.48
	Maternal parity	-4.66	3.36	-11.24	1.93	1.92	0.33
Foraging hourly rate	Infant age	0.08	0.06	-0.04	0.20	1.78	0.36
	Infant sex	0.35	0.20	-0.04	0.73	3.13	0.15
	Maternal parity	0.40	0.21	-0.01	0.80	3.59	0.12
Foraging bout duration	Infant age	0.09	0.12	0.05	0.12	20.33	**0.000**
	Infant sex	-0.01	0.03	-0.06	0.04	0.13	1.00
	Maternal parity	-0.01	0.04	-0.08	0.06	0.05	1.00

**Table 3:** Generalized Estimating Equations (GEEs) for correlations between feeding parameters of chimpanzees 6 months to 5 years old and infant age, sex, and maternal parity.

Infant feeding parameter	Infant or Maternal Characteristic	β	SE	95% Wald confidence interval		Hypothesis Test	
				Lower	Upper	Wald χ2 (df = 1)	*P* value
Percent time spent nursing	Infant age	-0.19	0.19	-0.56	0.18	0.98	0.64
	Infant sex	0.51	0.31	-0.10	1.12	2.70	0.20
	Maternal parity	-0.41	0.35	-1.10	0.28	1.36	0.49
Nursing hourly rate	Infant age	-0.09	0.06	-0.20	0.02	2.38	0.25
	Infant sex	0.01	0.09	-0.17	0.18	0.01	1.00
	Maternal parity	-0.01	0.11	-0.22	0.21	0.003	1.00
Nursing bout duration	Infant age	0.001	0.001	-0.001	0.003	0.52	0.95
	Infant sex	0.001	0.002	-0.004	0.005	0.10	1.00
	Maternal parity	-0.001	0.002	-0.006	0.003	0.24	1.00
Percent time spent foraging	Infant age	6.57	1.21	4.19	8.95	29.30	**0.000**
	Infant sex	1.07	2.42	-3.67	5.81	0.20	1.00
	Maternal parity	-5.51	2.14	-9.71	-1.32	6.64	**0.02**
Foraging hourly rate	Infant age	0.002	0.03	-0.05	0.05	0.01	1.00
	Infant sex	0.16	0.11	-0.05	0.38	2.17	0.28
	Maternal parity	-0.48	0.12	-0.71	-0.24	16.05	**0.000**
Foraging bout duration	Infant age	0.03	0.01	0.02	0.04	33.91	**0.000**
	Infant sex	-0.003	0.01	-0.03	0.02	0.07	1.00
	Maternal parity	0.000	0.01	-0.02	0.02	0.001	1.00

### Foraging

**Fig 4:**
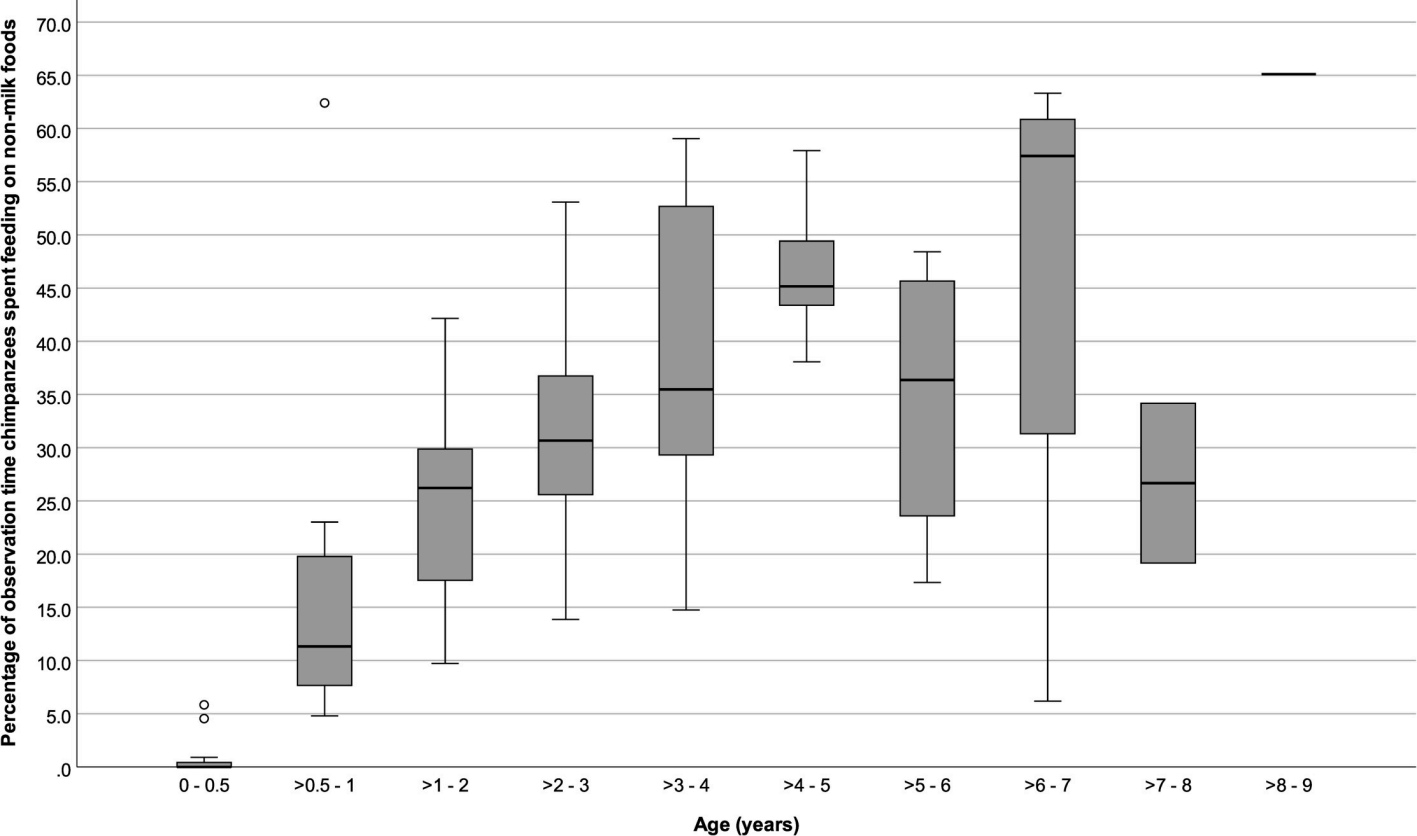
Percentage of focal observation time that immature chimpanzees spent foraging at different ages. Box plots show median values (solid horizontal lines), 25th and 75th percentile values (box boundaries), highest and lowest values (whiskers) and outliers (circles).

**Fig 5:**
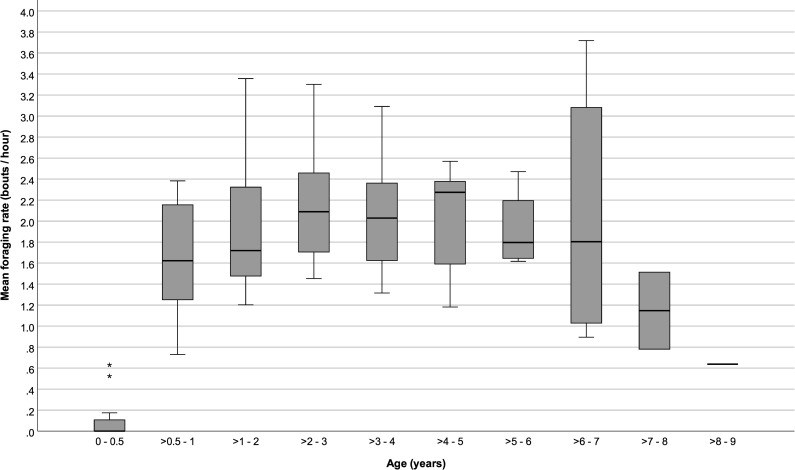
Average foraging rates of immature chimpanzees at different ages. Box plots show median values (solid horizontal lines), 25th and 75th percentile values (box boundaries), highest and lowest values (whiskers) and extremes (asterisks).

**Fig 6:**
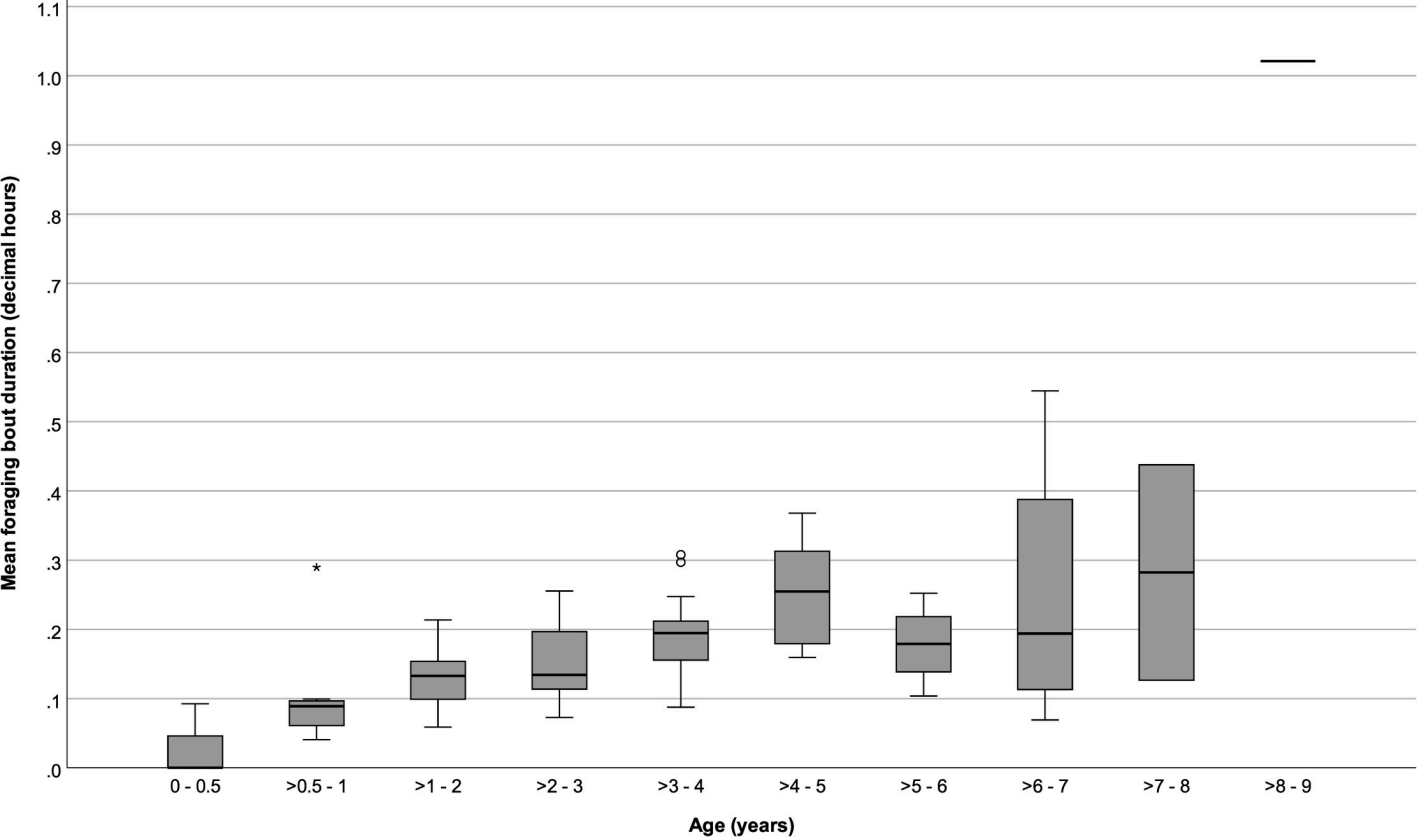
Average foraging bout durations of immature chimpanzees at different ages. Box plots show median values (solid horizontal lines), 25th and 75th percentile values (box boundaries), highest and lowest values (whiskers), outliers (circles), and extremes (asterisks).

Infants ≤ 6 months old spent 0.84 (SD: ± 1.81) % of their time foraging and feeding on non-milk foods and foraged on average for 0.11 (± 0.20) bouts per hour and for 1.48 (± 2.12) minutes per foraging bout. Infants > 6 months to ≤ 1 year old spent 17.18 (± 15.97) % of their time foraging and showed on average 1.64 (± 0.49) foraging bouts per hour and 5.87 (± 4.02) minutes per bout. From > 1 year old and onward, offspring foraging rates varied little, with a mean value of 1.99 (± 0.61) bouts per hour. However, the daily % time spent foraging and foraging bout durations were higher in older infants and ranged from 24.85 (± 8.13) % and 8.02 (± 2.52) minutes per foraging bout for infants > 1 to ≤ 2 years old, to a mean of 43.25 (± 21.78) % and 22.18 (± 18.61) minutes per foraging bout for individuals in age categories > 6 years old (Figs 4 to 6).

**Fig 7:**
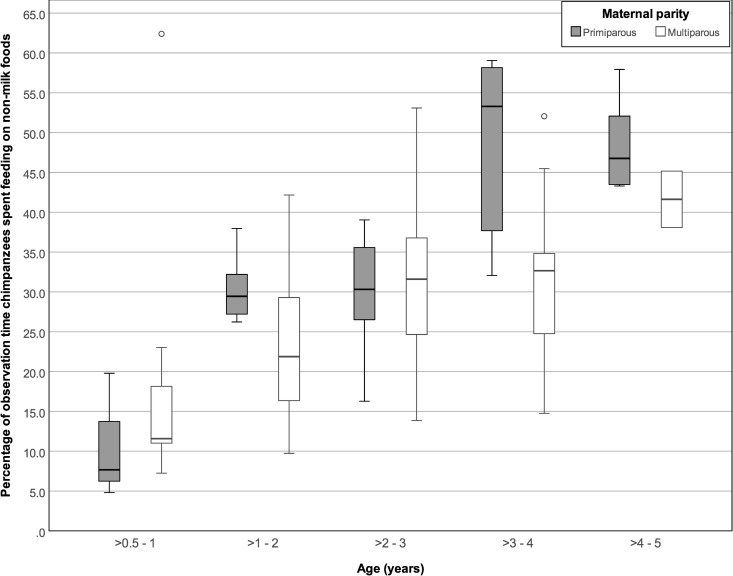
Percentage of focal observation time spent foraging by infants between 6 months and 5 years old of primiparous versus multiparous mothers. Box plots show median values (solid horizontal lines), 25th and 75th percentile values (box boundaries), highest and lowest values (whiskers) and outliers (circles).

Foraging rates did not vary significantly across age categories (GEE: 0 to ≤ 9 years old: *P* = 0.36, Table 2; > 6 months to ≤ 5 years old: *P* = 1.00, Table 3), but the daily % times spent foraging and average bout durations increased with age (0 to ≤ 9 years old: P < 0.001 and < 0.001, respectively; > 6 months to ≤ 5 years old: P < 0.001 and < 0.001, respectively). Among all individuals ≤ 9 years old, foraging parameters did not vary significantly with sex or maternal parity (Table 2). However, while foraging bout durations did not vary with maternal parity (P = 1.00) for infants 6 months to 5 years old, infants of primiparous females spent more time foraging (P < 0.05; Fig 7) and showed higher foraging hourly rates (P < 0.001) than infants of multiparous females (Table 3). Foraging parameters of infants 6 months to 5 years old did not vary significantly with infant sex.

## Discussion

### Infant feeding development and maternal lactation

The amount of time spent nursing and the rates and durations of nursing bouts were highest for newborns, 0 to 6 months old, which reflects the fact that milk is the only major source of energy during this time. This could reflect high nutritional needs to support peak postnatal infant growth rates directly after birth, like those of human babies [88]. It may also indicate that like other primates, chimpanzee infants take time to acquire the ability to nurse efficiently [12,87]. Newborns might need longer and more frequent nipple contacts than older infants to obtain enough milk. Indeed, newborns, who showed a head-bobbing reflex in search of their mothers’ nipples like human neonates do [89], often took time to latch and sometimes had difficulty remaining latched. Mothers might also have needed time to adjust, as newborns usually required extensive physical support to stay higher on the chest, which aided in latching.

Nursing parameters decreased with age but did not change significantly between 6 months and 5 years old. This could mean that the rate of milk synthesis did not vary greatly for mothers during this time, consistent with the fact that lactational performance (volume, quality, and rate of milk secretion) is largely controlled by the rate of mechanical stimulation of the nipple through suckling [8–13]. Age-invariant nursing behavior at Ngogo is consistent with data from chimpanzees at Gombe [21], but not with reports that nursing changed through infancy among chimpanzees at Kanyawara [22] and Mahale [23]. These inter-site differences suggest that despite the group-level clustering in nursing behavior that can be shown within chimpanzee communities, this behavior is susceptible to a degree of plasticity similar to that characterizing other developmental parameters, such as post-natal growth [90].

However, infant suckling efficiency might improve with age, even though the % time that infants suckled, and the rates and durations of nursing bouts varied little. If this was the case, older infants would obtain more milk during each nursing bout than younger infants [39]. Thus, maternal lactation effort would increase as infants aged, instead of remaining stable.

Another limitation to interpretation is our lack of data on night-time nursing, which may be common, as it is in humans [91]. If so, the nursing rates, durations and % times presented here underestimate the daily amount of nursing.

Ape infants take longer than one year to attain nutritional independence and are behaviorally weaned several years after birth (e.g. white-handed gibbons, *Hylobates lar*: 2.0 to 2.5 years; orangutan, *Pongo pygmaeus*: 7 years; gorilla, *Gorilla*: 2.8 to 4.6 years; *P*. *troglodytes*: 4 to 5 years; reviewed in [1] and [92]). Regular and prolonged nursing until behavioral weaning completion may be a pattern typical for hominoids, including humans [1,3,21,35,38], and cercopithecoids that can also take longer than a year to wean offspring behaviorally (e.g. Japanese macaques, *Macaca fuscata*: [33]; rhesus macaques, *Macaca mulatta*: [11]; yellow baboons, *Papio cynocephalus*: [18]; geladas, *Theropithecus gelada*: [34]). We previously used fecal stable nitrogen isotopes to show a gradual decrease in the relative contribution of milk to solid food in the diet of chimpanzee infants 1 to 4.5 years of age [4]. Given that nursing % times, bout rates and durations varied little through most of infancy, but independent foraging increased with age, we argue that infants were effectively leading their own gradual physiological weaning process. The decreasing relative contribution of milk to solid food in the diet seems to result from infants increasing their reliance on foods they procure independently rather from mothers gradually taking away lactational investment over the years.

While some infants sampled non-milk foods as early as 2 to 3 months after birth [4], these comprised a negligible proportion of the diet for at least the first 6 months, in line with expectations of infant dietary needs for apes and humans [1,3,38]. A notable difference between chimpanzees and humans is that by the time they are 6 months old, human infants need regular provisioning with complementary foods to sustain growth, while chimpanzees and other apes may regularly sample solid foods at this age, but do not require dietary supplementation with solid foods until months later [1,3,38,41,93]. Moreover, ape infants acquire most of their supplementary foods via their own foraging efforts, rather than via provisioning by others. Supplementation of maternal milk with solid foods has been predicted to take a primary dietary role at around 1 year of age in chimpanzees; this may be when infant energy needs surpass their mothers’ lactational capacities, and when infants thus must start meeting some of their own nutritional needs [1,3,38]. At Ngogo, this prediction is supported by a few lines of evidence. First, fecal stable isotope data indicated that the relative contribution of maternal milk to solid food in the diet began to decrease when infants were one year old [4], at which age infants start regularly incorporating solid foods in their diets. Second, foraging rates increased until chimpanzees were 1 year old, after which they remained stable, but foraging bout durations and % times continued to increase with age. This implies that by 1 year of age, infants were foraging as often as adult chimpanzees–which makes sense because chimpanzee infants foraged whenever their mothers did by this age [4,55,94]–but that they needed to forage longer per bout as they aged because they needed to supplement maternal milk with progressively more solid foods. Findings at other sites similarly showed that chimpanzee infants did not start feeding regularly on non-milk foods until close to 1 year of age, and that the overall time they spent foraging progressively increased through development [19,21–23].

Adult chimpanzees at Ngogo feed on average for 47% of their day [95]. Immature chimpanzees reached the adult-level 40 to 50% daily foraging times at around 5 years old at Kanyawara [22] and at 4.5 years old at Gombe [21]. Likewise, immature chimpanzees at Ngogo reached adult-level daily % foraging times by 4 to 5 years old, when they fed on non-milk foods for an average of 46.7 (SD ± 6.0) % of their day. After 5 years old, daily % foraging times varied greatly from one age category to another and between individuals, which could be due to the small number of chimpanzees in our sample after this age.

Most chimpanzees at Ngogo attained nutritional independence and were physiologically weaned by 4.5 years old, but a few continued comfort nursing for years after physiological weaning [4,6]. In the present study, three of four 5- to 6-year-olds and two of four 6- to 7-year-olds made nipple contacts during focal observations. Fecal stable isotope data established the presence of suckling with milk transfer for one of these individuals, while nipple contacts were for comfort, without milk transfer, for the others [4,6]. Our findings here showed that comfort nursing did not just involve occasional brief nipple contacts when infants checked mammary glands, but instead involved nipple contacts with measurable durations that occurred commonly, albeit at decreasing rates. The notion that some immature chimpanzees at Ngogo attained nutritional independence years before they stopped behaving as infants through continued nipple contacts accords with assessments of chimpanzee feeding development at other sites (Kanyawara: [41]; Mahale: [23]). Comfort nursing after physiological weaning may allow infants time to adjust to the changes that occur in the mother-infant social relationship with the infant-juvenile transition, and may result in behavioral weaning that is distinct from physiological weaning [6,96].

### Infant sex

We previously found that infant males were weaned later than females behaviorally, and likely physiologically, and that they had greater proportions of milk in their diets than same-aged females [6]. Two explanations for the greater proportions of milk in male diets could be that males generally nursed more often or longer per bout than females, or that they foraged less than females. However, our results were not consistent with either of these possibilities. Alternatively, males might have obtained more milk during each nursing bout than females [39]. Detailed data on infant cheek and jaw movements while suckling would help us estimate the amount of milk that infants obtained per bout [13,52]. Alternatively, immature males could be less efficient foragers than females; data on bite rates or the rate of food items ingested during foraging bouts could help discern this. Our results, however, are in line with findings from chimpanzees at Gombe that similarly showed no significant developmental differences between male and female infants in nursing and most foraging behaviors [21].

### Maternal parity

Infants of primiparous females foraged at higher rates and spent more time foraging than the infants of multiparous females. Primiparous females may produce lower quality milk and/or less milk than multiparous females [49,51,97–99] and may physiologically wean their infants later than their multiparous counterparts [100]. These differences could occur because primiparous females are less experienced mothers and new to lactation, while also often being physiologically immature and still developing while caring for their first infants [101–105]. Infants of primiparous mothers may need to compensate for lower milk quantities or quality by foraging more than infants of multipares do. However, the proportions of milk to solid food (mother-infant differences in fecal stable nitrogen isotopes) in the age specific diets of infants of primiparous and multiparous mothers were similar at Ngogo, as are inter-birth intervals of primiparous and multiparous females [6]. Thus, the higher foraging parameters found here for infants of primiparous mothers did not seem to lead to lower proportions of milk to solid food in their diets or to earlier attainment of nutritional independence compared to infants of multiparous mothers.

Females at Ngogo occasionally share substantial amounts of premasticated food with their infants, and the food most often shared is also the most commonly eaten by the chimpanzees (a fig, *Ficus mucuso*) [70,94]. We previously found that primiparous females shared premasticated food with their infants less often than multiparous females [94]. It may thus be that the infants of primiparous females needed to forage independently more to meet their dietary needs because their mothers shared less food with them.

### Future directions

Although we had a large dataset for a wild primate infant study, we had only enough data to make inferences about lactation and infant feeding development using mixed cross-sectional analytic methods. Collection of longitudinal data for individual mother-offspring pairs would make it possible to establish more precise estimates of population-level averages. Longitudinal, age-adjusted cohort data would also allow us to assess effects of temporal variation in the availability of foods other than milk. For example, analysis of dental barium levels of four orangutans (*P*. *abelii* and *P*. *pygmaeus*) throughout the entire infant period showed that orangutans can implement a cyclical pattern of lactation to accommodate changes in infant nursing during seasonal fluctuations in fruit availability [62]. We could not evaluate the effects of temporal variation in food availability on nursing. Ngogo is characterized by relatively high food abundance and relatively low variance in fruit availability [106–108] compared to other chimpanzee sites, and slight fluctuations in yearly food availability may have relatively little influence on lactation and infant feeding.

Comfort nursing deserves more investigation. It occurs after physiological weaning [4] but some suckling by infants who are not yet physiologically weaned might also have been non-nutritive. However, the consistency of nursing behavior during infancy supports the argument that nipple contacts were driven more by dietary than socio-emotional needs, given that specific quantities of milk would have been available at certain times due to the interaction between suckling stimuli and maternal lactational physiology [8–10,13].

### Implications for hominin evolution

Our aggregated findings are in line with the argument that chimpanzee mothers maintained a stable pattern of lactation effort, which may be evolutionarily more predictable for infants as they increasingly supplement their growing energy needs with non-milk foods and gradually attain the ability to feed independently [35,38]. As in humans [1,3,109–113], lactation effort in many apes might not change progressively over time, but instead might maintain a plateau through most of infancy [38]. Among the different patterns of lactation that could have characterized our early hominin ancestors, plateauing lactation is a good candidate for the common ancestral strategy [1,38]. Multi-year plateauing of lactation might have allowed hominoid infants time to attain the physiology and behavioral skills necessary for independent feeding, while also providing a steady and consistent nutritional base and buffering them from the negative effects of poor nutritional intake in early life [1,3,68]. Plateauing lactation would also have enabled females to maintain fixed, predictable levels of lactation effort through the course of infancy.

Compared to extant apes, humans have evolved a suite of adaptive characteristics associated with cooperative breeding that enabled females to have relatively short lactation periods for each offspring, and to invest in new infants well before previous offspring reached feeding independence, thus allowing for increased female reproductive rates [1,68,109,114–120]. Continued investigations of inter-population and inter-individual variation in infant feeding, weaning and lactation in chimpanzees and other apes will shed light on the evolutionary underpinnings that would have allowed early hominins to go from an ape-like model to the contemporary human form of infant feeding and maternal lactational investment.

## Supporting information

S1 Checklist Inclusivity in global research. (PDF)

S1 File Data used in analyses. (XLSX)

## References

[1] Sellen DW. Evolution of human lactation and complementary feeding: Implications for understanding contemporary cross-cultural variation. In: Goldberg G, Prentice A, Prentice A, Filteau S, Simondon K, editors. Breast-feeding: Early influences on later health. Dordrecht, Netherlands: Springer; 2009. p. 253–82. 10.1007/978-1-4020-8749-3_18 19227547

[2] Sellen DW. Evolution of infant and young child feeding: implications for contemporary public health. Annu Rev Nutr. 2007;27:123–48. doi: 10.1146/annurev.nutr.25.050304.092557 17666009

[3] Sellen DW. Lactation, complementary feeding, and human life history. In: Hawkes K, Paine RR, editors. The Evolution of Human Life History. Santa Fe, New Mexico: School of American Research Press; 2006.

[4] Bădescu I, Katzenberg MA, Watts DP, Sellen DW. A novel fecal stable isotope approach to determine the timing of age-related feeding transitions in wild infant chimpanzees. Am J Phys Anthropol. 2017;162(2):285–99. doi: 10.1002/ajpa.23116 27768227

[5] Martin P. The meaning of weaning. Anim Behav. 1984;32(4):1257–9.

[6] Bădescu I, Watts DP, Katzenberg MA, Sellen DW. Maternal lactational investment is higher for sons in chimpanzees. Behavioral Ecology and Sociobiology. 2022;76(3):44.

[7] Altmann J, Alberts SC. Growth rates in a wild primate population: ecological influences and maternal effects. Behavioral Ecology and Sociobiology. 2005;57(5):490–501.

[8] Howie PW, McNeilly AS. Effect of breast-feeding patterns on human birth intervals. Journal of Reproduction and Fertility. 1982;65:545–57. doi: 10.1530/jrf.0.0650545 7097656

[9] Gomendio M. Suckling behaviour and fertility in rhesus macaques (*Macaca mulatta*). Journal of Zoology, London. 1989;217:449–67.

[10] Johnson RL, Malik I, Berman CM. On the quantification of suckling intensity in primates. Am J Phys Anthropol. 1998;105:33–42. doi: 10.1002/(SICI)1096-8644(199801)105:1&lt;33::AID-AJPA4&gt;3.0.CO;2-E 9537926

[11] Gomendio M. Parent/offspring conflict and maternal investment in rhesus macaques. Animal Behaviour. 1991;42:993–1005.

[12] German RZ, Crompton AW, Hertwerck DW, Thexton AJ. Determinants of rhythm and rate in suckling. The Journal of Experimental Zoology. 1997;278:1–8. doi: 10.1002/(sici)1097-010x(19970501)278:1&lt;1::aid-jez1&gt;3.0.co;2-t 9136144

[13] Tanaka I. Parity-related differences in suckling behavior and nipple preference among free-ranging Japanese macaques. American Journal of Primatology. 1997;42:331–9. doi: 10.1002/(SICI)1098-2345(1997)42:4&lt;331::AID-AJP8&gt;3.0.CO;2-Y 9261514

[14] Charnov EL, Berrigan D. Why do female primates have such long lifespans and so few babies? Or life in the slow lane. Evolutionary Anthropology. 1993;1:191–4.

[15] Charnov EL. Evolution of life history variation among female mammals. Proceeding of the National Academy of Science of the United States of America. 1991;88(4):1134–7. doi: 10.1073/pnas.88.4.1134 1996315 PMC50971

[16] Ross C. Primate life histories. Evolutionary Anthropology. 1998;6(2):54–63.

[17] Pusey AE. Magnitude and sources of variation in female reproductive performance. In: Mitani JC, Call J, Kappeler PM, Palombit RA, Silk JB, editors. The Evolution of Primate Societies. 1st ed. Chicago, United States of America: The University of Chicago Press; 2012. p. 343–66.

[18] Altmann J. Baboon mothers and infants. Chicago: The University of Chicago Press; 1980. 242 p.

[19] Hiraiwa-Hasegawa M. A note on the ontogeny of feeding. In: Nishida T, editor. The chimpanzees of the Mahale Mountains Sexual and life history strategies. Tokyo: University of Tokyo Press; 1990. p. 277–83.

[20] Fletcher AW. Development of infant independence from the mother in wild mountain gorillas. In: Robbins MM, Sicotte P, Stewart KJ, editors. Mountain gorillas: Three decades of research at Karisoke. New York, USA: Canbridge University Press; 2001. p. 153–83.

[21] Lonsdorf EV, Markham AC, Heintz MR, Anderson KE, Ciuk DJ, Goodall J, et al. Sex differences in wild chimpanzee behavior emerge during infancy. PLOS ONE. 2014;9(6):e99099. doi: 10.1371/journal.pone.0099099 24911160 PMC4049619

[22] Bray J, Emery Thompson M, Muller MN, Wrangham RW, Machanda ZP. The development of feeding behavior in wild chimpanzees (Pan troglodytes schweinfurthii). Am J Phys Anthropol. 2018;165(1):34–46. doi: 10.1002/ajpa.23325 28949015 PMC5739981

[23] Matsumoto T. Developmental changes in feeding behaviors of infant chimpanzees at Mahale, Tanzania: Implications for nutritional independence long before cessation of nipple contact. Am J Phys Anthropol. 2017;163(2):356–66. doi: 10.1002/ajpa.23212 28319268

[24] Forster S, Cords M. Development of mother-infant relationships and infant behavior in wild blue monkeys (*Cercopithecus mitis stuhlmanni*). In: Glenn ME, Cords M, editors. The guenons: Diversity and adaptation in African monkeys. US: Springer; 2002. p. 245–72.

[25] Barrett L, Henzi PS, Lycett JE. Whose life is it anyway? Maternal investment, developmental trajectories, and life history strategies in baboons. In: Swedell L, Leigh SR, editors. Reproduction and fitness in baboons: Behavioral, ecological, and life history perspectives. London, UK: Kluwer Academic Press; 2006. p. 199–224.

[26] Nicolson NA. Weaning and the development of independence in olive baboons. Cambridge, MA: Harvard University; 1982.

[27] Lee PC. Nutrition, fertility and maternal investment in primates. Journal of Zoology, London. 1987;213:409–22.

[28] Stewart KJ. Suckling and lactational anoestrus in wild gorillas (*Gorilla gorilla*). Journal of Reproduction and Fertility. 1988;83:627–34. doi: 10.1530/jrf.0.0830627 3411555

[29] Clark CB. A preliminary report on weaning among chimpanzees of the Gombe National Park, Tanzania. In: Chevalier-Skolonikoff S, Poirier FE, editors. Primate Bio-social Development. New York: Garland Press; 1977. p. 235–60.

[30] Langer P. The phases of maternal investment in eutherian mammals. Zoology (Jena). 2008;111(2):148–62. doi: 10.1016/j.zool.2007.06.007 18222662

[31] Lee PC. Growth and investment in hominin life history evolution: Patterns, processes, and outcomes. Int J Primatol. 2012;33(6):1309–31.

[32] Humphrey LT. Weaning behaviour in human evolution. Seminars in cell & developmental biology. 2010;21(4):453–61. doi: 10.1016/j.semcdb.2009.11.003 19914386

[33] Worlein JM, Eaton GG, Johnson DF, Glick BB. Mating season effects on mother-infant conflict in Japanese macaques, Macaca fuscata. Animal Behaviour. 1988;36(5):1472–81.

[34] Barrett L, Dunbar RIM, Dunbar P. Mother-infant contact as contingent behaviour in gelada baboons. Anim Behav. 1995;49:805–10.

[35] van Noordwijk MA, Willems EP, Utami Atmoko SS, Kuzawa CW, van Schaik CP. Multi-year lactation and its consequences in Bornean orangutans (*Pongo pygmaeus wurmbii*). Behavioral Ecology and Sociobiology. 2013;67:805–14.

[36] Lee PC. The meanings of weaning: growth, lactation, and life history. Evol Anthropol. 1997;5:87–96.

[37] Kennedy G. From the ape’s dilemma to the weanling’s dilemma: early weaning and its evolutionary context. J Hum Evol. 2005;48(2):123–45. doi: 10.1016/j.jhevol.2004.09.005 15701527

[38] van Noordwijk MA, Kuzawa CW, Van Schaik CP. The evolution of the patterning of human lactation: A comparative perspective. Evol Anthropol. 2013;22(5):202–12. doi: 10.1002/evan.21368 24166921

[39] Cameron EZ. Is suckling behaviour a useful predictor of milk intake? A review. Animal Behaviour. 1998;56(521–532). doi: 10.1006/anbe.1998.0793 9784199

[40] Hinde K, Milligan LA. Primate milk: proximate mechanisms and ultimate perspectives. Evol Anthropol. 2011;20(1):9–23. doi: 10.1002/evan.20289 22034080

[41] Smith TM, Machanda Z, Bernard AB, Donovan RM, Papakyrikos AM, Muller MN, et al. First molar eruption, weaning, and life history in living wild chimpanzees. Proceedings of the National Academy of Sciences of the United States of America. 2013;110(8):2787–91. doi: 10.1073/pnas.1218746110 23359695 PMC3581971

[42] Borries C, Lu A, Ossi-Lupo K, Larney E, Koenig A. The meaning of weaning in wild Phayre’s leaf monkeys: last nipple contact, survival, and independence. Am J Phys Anthropol. 2014;154(2):291–301. doi: 10.1002/ajpa.22511 24615436

[43] Stanton MA, Lonsdorf EV, Murray CM, Pusey AE. Consequences of maternal loss before and after weaning in male and female wild chimpanzees. Behavioral Ecology and Sociobiology. 2020;74(2).

[44] Boesch C, Bole C, Eckhardt N, Boesch H. Altruism in forest chimpanzees: the case of adoption. PLoS One. 2010;5(1):e8901. doi: 10.1371/journal.pone.0008901 20111704 PMC2811728

[45] Roberts SB, Cole TJ, Coward WA. Lactational performance in relation to energy intake in the baboon. The American Journal of Clinical Nutrition. 1985;41:1270–6. doi: 10.1093/ajcn/41.6.1270 4003332

[46] Prentice AM, Prentice A. Evolutionary and evironmental influences on human lactation. Proceedings of the Nutrition Society. 1995;54:391–400. doi: 10.1079/pns19950008 8524886

[47] Tardif SD, Power M, Oftedal OT, Power RA, Layne DG. Lactation, maternal behavior and infant growth in common marmoset monkeys (Callithrix jacchus): effects of maternal size and litter size. Behavioral Ecology and Sociobiology. 2001;51(1):17–25.

[48] Hinde K. Milk composition varies in relation to the presence and abundance of Balantidium coli in the mother in captive rhesus macaques (Macaca mulatta). Am J Primatol. 2007;69(6):625–34. doi: 10.1002/ajp.20373 17245767

[49] Hinde K, Power ML, Oftedal OT. Rhesus macaque milk: magnitude, sources, and consequences of individual variation over lactation. Am J Phys Anthropol. 2009;138(2):148–57. doi: 10.1002/ajpa.20911 18711734 PMC2615798

[50] Stanton MA, Lonsdorf EV, Pusey AE, Goodall J, Murray CM. Maternal Behavior by Birth Order in Wild Chimpanzees (Pan troglodytes): Increased Investment by First-Time Mothers. Curr Anthropol. 2014;55(4):483–9. doi: 10.1086/677053 25328164 PMC4197843

[51] Hinde K. Richer milk for sons but more milk for daughters: Sex-biased investment during lactation varies with maternal life history in rhesus macaques. American journal of human biology: the official journal of the Human Biology Council. 2009;21(4):512–9. doi: 10.1002/ajhb.20917 19384860

[52] Tanaka I. Three phases of lactation in free-ranging Japanese macaques. Animal Behaviour. 1992;44:129–39.

[53] Tilden CD, Oftedal OT. Milk composition relfects pattern of maternal care in prosimian primates. American Journal of Primatology. 1997;41:195–211. doi: 10.1002/(SICI)1098-2345(1997)41:3&lt;195::AID-AJP3&gt;3.0.CO;2-S 9057965

[54] Whittier CA, Milligan LA, Nutter FB, Cranfield MR, Power ML. Proximate composition of milk from free-ranging mountain gorillas (Gorilla beringei beringei). Zoo Biol. 2011;30(3):308–17. doi: 10.1002/zoo.20363 21061295

[55] Bădescu I, Watts DP, Katzenberg MA, Sellen DW. Alloparenting is associated with reduced maternal lactation effort and faster weaning in wild chimpanzees. Royal Society open science. 2016;3(11):160577. doi: 10.1098/rsos.160577 28018647 PMC5180145

[56] Fairbanks LA. Reciprocal benefits of allomothering for female vervet monkeys. Animal Behaviour. 1990;40(3):553–62.

[57] Gomendio M. The influence of maternal rank and infant sex on maternal investment trends in Rhesus macaques: Birth sex ratios, inter-birth intervals and suckling patterns. Behavioral Ecology and Sociobiology. 1990;27(5):365–75.

[58] Quinlan RJ, Quinlan MB, Flinn MV. Local resource enhancement and sex-biased breastfeeding in a Caribbean community. Curr Anthrop. 2005;46:471–80.

[59] Hinde K. First-time macaque mothers bias milk composition in favor of sons. Current biology: CB. 2007;17(22):R958–9. doi: 10.1016/j.cub.2007.09.029 18029247

[60] de Passillé AMB, Rushen J. Calves’ behaviour during nursing is affected by feeding motivation and milk availability. Applied Animal Behaviour Science. 2006;101(3–4):264–75.

[61] Drewett RF, Woolridge M. Sucking patterns of human babies on the breast. Early Human Development. 1979;3/4:315–20. doi: 10.1016/0378-3782(79)90042-2 535550

[62] Smith TM, Austin C, Hinde K, Vogel ER, Arora M. Cyclical nursing patterns in wild orangutans. Sci Adv. 2017;3(e1601517):1–8. doi: 10.1126/sciadv.1601517 28560319 PMC5435413

[63] Watts DP. Observations on the ontogeny of feeding behavior in mountain gorillas (*Gorilla gorilla beringei*). Am J Primatol. 1985;8:1–10. doi: 10.1002/ajp.1350080102 31986827

[64] Lonsdorf EV, Eberly LE, Pusey AE. Sex differences in learning in chimpanzees. Nature. 2004;428(6984):715–6. doi: 10.1038/428715a 15085121

[65] Jaeggi AV, van Noordwijk MA, van Schaik CP. Begging for information: mother-offspring food sharing among wild Bornean orangutans. Am J Primatol. 2008;70(6):533–41. doi: 10.1002/ajp.20525 18186082

[66] Rapaport LG, Brown GR. Social influences on foraging behavior in young nonhuman primates: Learning what, where, and how to eat. Evol Anthropol. 2008;17:189–201.

[67] Boinski S, Fragaszy DM. The ontogeny of foraging in squirrel monkeys, Saimiri oerstedi. Animal Behaviour. 1989;37:415–28.

[68] Hrdy SB. Mothers and Others: The Evolutionary Origins of Mutual Understanding. Cambridge, Massachusetts: The Belknap Press of Harvard University Press; 2009.

[69] Lwanga JS. Forest succession in Kibale National Park, Uganda: Implications for forest restoration and management. Afr J Ecol. 2003;41:9–22.

[70] Watts DP, Potts KB, Lwanga JS, Mitani JC. Diet of chimpanzees (*Pan troglodytes schweinfurthii*) at Ngogo, Kibale National Park, Uganda, 1. Diet composition and diversity. Am J Primatol. 2012;74(2):114–29. doi: 10.1002/ajp.21016 22109938

[71] Chapman CA, Chapman LJ, Struhsaker TT, Zanne AE, Clark CJ, Poulsen JR. A long-term evaluation of fruiting phenology: importance of climate change. J Trop Ecol. 2005;21(1):31–45.

[72] Sandel AA, Watts DP. Lethal Coalitionary Aggression Associated with a Community Fission in Chimpanzees (*Pan troglodytes*) at Ngogo, Kibale National Park, Uganda. International Journal of Primatology. 2021;42(1):26–48. doi: 10.1007/s10764-020-00185-0 34267410 PMC8277110

[73] Watts DP, Potts KB, Lwanga JS, Mitani JC. Diet of chimpanzees (*Pan troglodytes schweinfurthii*) at Ngogo, Kibale National Park, Uganda, 2. Temporal variation and fallback foods. Am J Primatol. 2012;74(2):130–44. doi: 10.1002/ajp.21015 22125130

[74] Watts DP, Mitani JC. Hunting and prey switching by chimpanzees (*Pan troglodytes schweinfurthii*) at Ngogo. Int J Primatol. 2015;36(4):728–48.

[75] Watts DP, Mitani JC. Hunting behavior of chimpanzees at Ngogo, Kibale National Park, Uganda. Int J Primatol. 2002;23(1):1–28.

[76] Watts DP, Mitani J. Hunting and meat sharing by chimpanzees at Ngogo, Kibale National Park, Uganda. In: Boesch C, Hohmann G, Marchant LF, editors. Behavioural Diversity in Chimpanzees and Bonobos. Cambridge, United Kingdom: Cambridge University Press; 2002. p. 231–43.

[77] Wakefield ML. Grouping Patterns and Competition Among Female Pan troglodytes schweinfurthii at Ngogo, Kibale National Park, Uganda. International Journal of Primatology. 2008;29(4):907–29.

[78] Wakefield ML. Social dynamics among females and their influence on social structure in an East African chimpanzee community. Animal Behaviour. 2013;85(6):1303–13.

[79] Langergraber K, Mitani JC, Vigilant L. Kinship and social bonds in female chimpanzees (*Pan troglodytes*). American Journal of Primatology. 2009;71:1–12. 19475543 10.1002/ajp.20711

[80] Goodall J. The chimpanzees of Gombe. Cambridge, MA: Harvard University Press; 1986.

[81] Altmann J. Observational study of behavior: Sampling methods. Behaviour. 1974;49(3/4):227–67. doi: 10.1163/156853974x00534 4597405

[82] Liang K, Zeger SL. Longitudinal data analysis using generalized linear models. Biometrika. 1986;73(1):13–22.

[83] Ghisletta P, Spini D. An introduction to Generalized Estimating Equations and an application to assess selectivity effects in a longitudinal study on very old individuals. J Ed Behav St. 2004;29(421–437).

[84] Zuur AF, Ieno EN, Walker NJ, Saveliev AA, Smith GM. Mixed Effects Models and Extensions in Ecology with R. New York: Springer Science+Business Media; 2009.

[85] Katzenberg MA, Pfeiffer S. Nitrogen isotope evidence for weaning age in a 19th century Canadian skeletal sample. In: Grauer A, editor. Bodies of evidence: reconstructing history through skeletal analysis. New York: Wiley-Liss; 1995. p. 221–35.

[86] Prowse TL, Saunders SR, Schwarcz HP, Garnsey P, Macchiarelli R, Bondioli L. Isotopic and dental evidence for infant and young child feeding practices in an imperial Roman skeletal sample. Am J Phys Anthropol. 2008;137(3):294–308. doi: 10.1002/ajpa.20870 18615573

[87] Paul K, Dittrichova J, Papousek H. Infant feeding behavior: Development in patterns and motivation. Developmental Psychobiology. 1996;29(7):563–76. doi: 10.1002/(SICI)1098-2302(199611)29:7&lt;563::AID-DEV2&gt;3.0.CO;2-S 8911772

[88] Eveleth PB, Tanner JM. Worldwide variation in human growth. Cambridge: Cambridge University Press; 1976.

[89] Colson SD, Meek JH, Hawdon JM. Optimal positions for the release of primitive neonatal reflexes stimulating breastfeeding. Early Hum Dev. 2008;84(7):441–9. doi: 10.1016/j.earlhumdev.2007.12.003 18243594

[90] Kuzawa CW, Bragg JM. Plasticity in human life history strategy Implications for contemporary human variation and the evolution of genus *Homo*. Curr Anthrop. 2012;53(S6):S369–S82.

[91] Ball H, Klingaman K. Breastfeeding and mother-infant sleep proximity. Evolutionary medicine and health. 2008:226–41.

[92] Alvarez HP. Grandmother hypothesis and primate life histories. Am J Phys Anthropol. 2000;113:435–50. doi: 10.1002/1096-8644(200011)113:3&lt;435::AID-AJPA11&gt;3.0.CO;2-O 11042542

[93] Fahy GE, Richards MP, Fuller BT, Deschner T, Hublin JJ, Boesch C. Stable nitrogen isotope analysis of dentine serial sections elucidate sex differences in weaning patterns of wild chimpanzees (*Pan troglodytes*). Am J Phys Anthropol. 2014;153(4):635–42. doi: 10.1002/ajpa.22464 24395019

[94] Bădescu I, Sicotte P, Sandel AA, Desruelle KJ, Curteanu C, Watts DP, et al. Premasticated food transfer by wild chimpanzee mothers with their infants: Effects of maternal parity, infant age and sex, and food properties. J Hum Evol. 2020;143:102794. doi: 10.1016/j.jhevol.2020.102794 32371289

[95] Potts KB, Watts DP, Wrangham RW. Comparative Feeding Ecology of Two Communities of Chimpanzees (Pan troglodytes) in Kibale National Park, Uganda. International Journal of Primatology. 2011;32(3):669–90.

[96] Bădescu. The attainment of independence from the mother in primate infants and its implications for the evolution of cooperative breeding in hominins. In: Luef EM, Marin MM, editors. The talking species: Perspectives on the evolutionary, neuronal and cultural foundations of language. Graz, Austria: Unipress Graz Verlag; 2018. p. 165–91.

[97] Künkele J, Kenagy GJ. Inefficiency of Lactation in Primiparous Rats: The Costs of First Reproduction. Physiological Zoology. 1997;70(5):571–7. doi: 10.1086/515862 9279924

[98] Künkele J. Does primiparity affect the efficiency of converting energy to offspring production in the guinea-pig? Canadian Journal of Zoology. 2000;78(2):300–6.

[99] Motil KJ, Kertz B, Thotathuchery M. Lactational performance of adolescent mothers shows preliminary differences from that of adult women. Journal of Adolescent Health. 1997;20(6):442–9. doi: 10.1016/S1054-139X(97)00036-0 9178081

[100] Eckardt W, Fawcett K, Fletcher AW. Weaned age variation in the Virunga mountain gorillas (Gorilla beringei beringei): influential factors. Behavioral Ecology and Sociobiology. 2016;70(4):493–507.

[101] Stearns SC. The evolution of life histories. Oxford, UK: Oxford University Press; 1992.

[102] Clutton-Brock T. The evolution of parental care. Princeton: Princeton University Press; 1991.

[103] Lipkin EW, Aumann CA, Newell-Morris LL. Evidence for common controls over inheritance of bone quantity and body size from segregation analysis in a pedigreed colony of nonhuman primates (Macaca nemestrina). Bone. 2001;29(3):249–57. doi: 10.1016/s8756-3282(01)00508-7 11557369

[104] Cerroni AM, Tomlinson GA, Turnquist JE, Grynpas MD. Effect of parity on bone mineral density in female rhesus macaques from Cayo Santiago. Amer J Phys Anthrop. 2003;121(3):252–69. doi: 10.1002/ajpa.10238 12772213

[105] Robbins AM, Robbins MM, Gerald-Steklis N, Steklis HD. Age-related patterns of reproductive success among female mountain gorillas. Amer J Phys Anthrop. 2006;131(4):511–21. doi: 10.1002/ajpa.20474 16941601

[106] Wood BM, Watts DP, Mitani JC, Langergraber KE. Favorable ecological circumstances promote life expectancy in chimpanzees similar to that of human hunter-gatherers. J Hum Evol. 2017;105:41–56. doi: 10.1016/j.jhevol.2017.01.003 28366199 PMC5526083

[107] Potts KB, Baken E, Ortmann S, Watts DP, Wrangham RW. Variability in Population Density Is Paralleled by Large Differences in Foraging Efficiency in Chimpanzees (Pan troglodytes). International Journal of Primatology. 2015;36(6):1101–19.

[108] Potts KB. Nutritional ecology and reproductive output in female chimpanzees (*Pan troglodytes*): variation among and within populations. In: Clancy KBH, Hinde K, Rutherford JN, editors. Building babies: Primate development in proximate and ultimate perspective. New York: Springer Science+Business Media; 2013. p. 83–100.

[109] Kaplan H, Hill K, Lancaster J, Hurtado AM. A theory of human life history evlution: Diet, intelligence, and longevity. Evolutionary Anthropology. 2000;9(4):156–85.

[110] Butte NF. Energy requirements of infants. Public Health Nutrition. 2005;8(7A):953–67. doi: 10.1079/phn2005790 16277814

[111] Butte NF, King JC. Energy requirements during pregnancy and lactation. Public Health Nutrition. 2005;8(7A):110–1027. doi: 10.1079/phn2005793 16277817

[112] Piperata BA, Dufour DL. Diet, energy expenditure, and body composition of lactating Ribeirinha women in the Brazilian Amazon. American journal of human biology: the official journal of the Human Biology Council. 2007;19(5):722–34. 17657725 10.1002/ajhb.20628

[113] Piperata BA. Variation in maternal strategies during lactation: the role of the biosocial context. American journal of human biology: the official journal of the Human Biology Council. 2009;21(6):817–27. doi: 10.1002/ajhb.20898 19360702

[114] Hawkes K, O’Connell JF, Jones NG, Alvarez H, Charnov EL. Grandmothering, menopause, and the evolution of human life histories. Proceedings of the National Academy of Sciences of the United States of America. 1998;95(3):1336–9. doi: 10.1073/pnas.95.3.1336 9448332 PMC18762

[115] Kramer KL. Children’s Help and the Pace of Reproduction: Cooperative Breeding in Humans. Evolutionary Anthropology: Issues, News, and Reviews. 2005;14(6):224–37.

[116] Kramer KL. Cooperative Breeding and its Significance to the Demographic Success of Humans. Annu Rev Anthrop. 2010;39(1):417–36.

[117] Hawkes K, Coxworth JE. Grandmothers and the evolution of human longevity: a review of findings and future directions. Evol Anthropol. 2013;22(6):294–302. doi: 10.1002/evan.21382 24347503

[118] Bogin B, Bragg J, Kuzawa C. Humans are not cooperative breeders but practice biocultural reproduction. Ann Hum Biol. 2014;41(4):368–80. doi: 10.3109/03014460.2014.923938 24932750

[119] Hawkes K. Primate sociality to human cooperation. Why us and not them? Hum Nat. 2014;25(1):28–48. doi: 10.1007/s12110-013-9184-x 24307447

[120] Kramer KL, Otarola-Castillo E. When mothers need others: The impact of hominin life history evolution on cooperative breeding. J Hum Evol. 2015;84:16–24. doi: 10.1016/j.jhevol.2015.01.009 25843884

